# Reshaping the phonon energy landscape of nanocrystals inside a terahertz plasmonic nanocavity

**DOI:** 10.1038/s41467-018-03120-3

**Published:** 2018-02-22

**Authors:** Xin Jin, Andrea Cerea, Gabriele C. Messina, Andrea Rovere, Riccardo Piccoli, Francesco De Donato, Francisco Palazon, Andrea Perucchi, Paola Di Pietro, Roberto Morandotti, Stefano Lupi, Francesco De Angelis, Mirko Prato, Andrea Toma, Luca Razzari

**Affiliations:** 1 INRS Énergie, Matériaux et Télécommunications, 1650 Blvd Lionel Boulet, J3X 1S2 Varennes, QC Canada; 2 Istituto Italiano di Tecnologia, via Morego 30, 16163 Genova, Italy; 3 Dipartimento di Informatica, Bioingegneria, Robotica e Ingegneria dei Sistemi (DIBRIS), Università degli Studi di Genova, via Balbi 5, 16126 Genova, Italy; 4 Elettra - Sincrotrone Trieste S.C.p.A, AREA Science Park, S.S. 14 km163.5, Trieste, 34149 Italy; 5 National Research University of Information Technologies, Mechanics and Optics, 199034 Saint Petersburg, Russia; 6 Institute of Fundamental and Frontier Sciences, University of Electronic Science and Technology of China, Chengdu, Sichuan 610054 China; 7 CNR-IOM and Dipartimento di Fisica, Università di Roma “La Sapienza”, Piazzale A. Moro 2, Roma, I-00185 Italy

## Abstract

Phonons (quanta of collective vibrations) are a major source of energy dissipation and drive some of the most relevant properties of materials. In nanotechnology, phonons severely affect light emission and charge transport of nanodevices. While the phonon response is conventionally considered an inherent property of a nanomaterial, here we show that the dipole-active phonon resonance of semiconducting (CdS) nanocrystals can be drastically reshaped inside a terahertz plasmonic nanocavity, via the phonon strong coupling with the cavity vacuum electric field. Such quantum zero-point field can indeed reach extreme values in a plasmonic nanocavity, thanks to a mode volume well below *λ*^3^/10^7^. Through Raman measurements, we find that the nanocrystals within a nanocavity exhibit two new “hybridized” phonon peaks, whose spectral separation increases with the number of nanocrystals. Our findings open exciting perspectives for engineering the optical phonon response of functional nanomaterials and for implementing a novel platform for nanoscale quantum optomechanics.

## Abstract (web summary)

Here the authors show that the dipole-active phonon resonance of semiconducting nanocrystals can be hybridized by a strongly concentrated terahertz vacuum field of a plasmonic nanocavity, thus achieving strong plasmon–phonon coupling even in the absence of direct terahertz illumination.

## Introduction

Strong radiation-matter coupling in optical cavities has been the subject of extensive research in the last two decades and has led to fundamental discoveries in, e.g., cavity quantum electrodynamics^1,2^ and solid-state Bose–Einstein condensation^3^. Traditionally, electronic excitations in atoms or condensed matter systems (e.g., excitonic resonances) have been investigated, exploiting conventional Fabry–Pérot cavities^1,3–6^, as well as photonic–crystal^2,7^ and plasmonic resonators^8–11^. In particular, hybridized excitons in optical cavities have been found to acquire distinctive properties, such as a significantly reduced lifetime^2^, and have been the foundation of a novel form of lasing mechanism featuring extremely low threshold, called polariton lasing^4,5^.

Very recently, it has been shown that also vibrational transitions associated with chemical bonds in organic molecules can be used to achieve strong light-matter coupling^12–17^. Hybridization of specific molecular vibrations has been obtained by filling Fabry–Pérot microcavities featuring sharp infrared resonances with either solid layers^12,13^ or liquids^14–16^ containing the targeted molecules. These latest studies have significant implications in many fields, for instance in the advanced control of chemical reactions^14,16^ as well as for the exploration of cavity optomechanics at room temperature^17^. Achieving vibrational strong coupling in nanoscale volumes promises to further extend the reach of these investigations, for example, facilitating the characterization of the quantum nature of such interactions, by drastically limiting the number of involved “vibrational matter oscillators”. Indeed, hybrid quantum systems, composed of two or more quantum entities with a different physical nature, are bound to shape the next generation of quantum technologies, offering an integrated solution for performing multiple tasks (e.g., quantum information transmission, processing and storage)^18^.

**Fig. 1:**
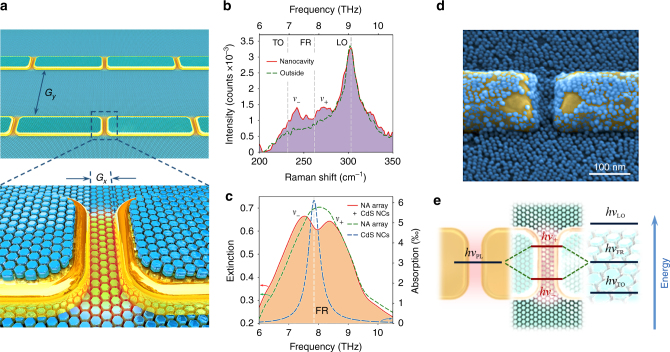
NC phonon hybridization inside a plasmonic nanocavity. **a** Graphical representation of a plasmonic nanoantenna array covered with a monolayer of NCs. **b** Raman spectra collected in a nanocavity region (solid red line) and just outside the nanocavity on the silicon substrate (green dashed line) for a sample covered with an NC monolayer; length of nanoantennas *L* = 5.75 μm. **c** Experimental THz extinction spectra of the array featuring nanoantennas of length *L* = 5.75 μm with (solid red line) and without (green dashed line) an NC monolayer over its surface; blue dashed line: absorption spectrum of a single layer of NCs (NA array: nanoantenna array). **d** Scanning electron microscope (SEM) image of a nanocavity region covered with a monolayer of NCs. **e** Energy diagram exemplifying the plasmon–phonon resonance hybridization

In this work, we demonstrate plasmon–phonon hybridization^19–22^ in a nanoscale system strongly coupled with the terahertz (THz) vacuum field. This is achieved by combining the extreme radiation confinement properties of THz plasmonic nanocavities^23^ with the marked dipole-active phonon resonance typical of polar nanocrystals (NCs)^24^ (see pictorial representation in Fig. 1a and energy hybridization scheme in Fig. 1e). In such plasmonic nanocavities, the THz vacuum electric field *E*_vac_ (i.e., the field associated with the quantum zero-point energy of the cavity resonant mode) can reach extraordinary high values, since it scales with the cavity mode volume *V*_mod_ as: $\left| {E_{{\mathrm{vac}}}} \right| \propto \sqrt {1/V_{\bmod }}$. As for the phononic system, we employ cadmium sulphide (CdS) NCs, a type of nanomaterial that has been long investigated for its promising applications in optoelectronics^25^.

## Results

### Phonon resonance hybridization in a THz plasmonic nanocavity

Colloidal, well-monodispersed CdS NCs with an average size of 10 nm were chemically synthesized using an established method (see Supplementary Note 2 for details). Such NCs are endowed with three main phonon lines: the transverse optical (TO) and longitudinal optical (LO) phonon modes, which are also characteristic of bulk crystalline CdS, as well as a Fröhlich (FR) optical phonon resonance, typical of nanoparticles^24^, which lies between the TO and LO lines. These three lines are identified in Fig. 1b, over the Raman spectrum of a single layer of CdS NCs deposited on a silicon substrate (dashed green curve: *v*_TO _≈ 6.9 THz, *v*_LO _≈ 9.1 THz, *v*_FR _≈ 7.85 THz). As can be seen in this figure, the FR mode corresponds to a low-frequency tail of the NC main peak (LO phonon mode) in Raman measurements. Conversely, being the FR phonon dipole active, it can be clearly observed in direct THz absorption measurements. This is shown in Fig. 1c, where the THz absorption lineshape of the monolayer of NCs is depicted (blue dashed curve), as extracted from THz transmission measurements (see Supplementary Note 6 and Supplementary  Figure 10 for the exact procedure). Such dipole-active transition is thus the target of our investigation, since it can be effectively coupled to electromagnetic radiation. To this end, we prepared an array of gold plasmonic nanoantennas (covering an area of 200 × 200 μm^2^) separated by narrow gaps (*G*_*x*_ = 30 nm in Fig. 1a) along their long-axis direction. The nanoantennas are 200 nm wide, 55 nm high and their spacing along their short-axis direction is *G*_*y*_ = 8.5 μm (Fig. 1a). By properly tuning the length *L* of these nanoantennas, it is possible to align their plasmonic resonance (dashed green curve in Fig. 1c) to the FR line of the NCs. This is obtained for *L* = 5.75 μm. The fabricated array (see Supplementary Note 1 for fabrication details) was then spin-coated with a solution containing the CdS NCs, resulting in a uniform monolayer that well covers the sample surface, even within the antenna nanocavities (see scanning electron microscope (SEM) image in Fig. 1d). THz transmission measurements, performed using a Fourier-transform THz microscope coupled to synchrotron light (ELETTRA Trieste, SISSI beamline^26^, see Supplementary Note 4 for details), evidence the splitting of the nanoantenna resonance into two peaks, identified as *v*_−_ and *v*_+_ in Fig. 1c (red curve), hinting at the  onset of a plasmon–phonon strong coupling. In true strong coupling conditions, one expects the hybridization to also occur without the need of any THz illumination, since the two oscillations should be coupled strongly just by means of the vacuum field^27^. To confirm this, we performed a micro-Raman characterization of the NCs located in the plasmonic nanocavity regions (see Supplementary Note 5 for experimental details). Indeed, Raman spectroscopy, by simply monitoring the frequency of visible light inelastically scattered by the investigated system, can retrieve the phonon response without recurring to a direct THz illumination. An example of these measurements is shown in Fig. 1b (red curve). As can be seen, two new peaks arise in the Raman spectrum at the two sides of the FR resonance, providing clear evidence of the creation of a new hybrid nanosystem with phonon properties that no longer belong to the original nanomaterial. Such modification of the Raman response is indeed remarkable, especially considering the fact that the focal spot of the micro-Raman system we employed (beam area: ~1.39 μm^2^) is more than 200 times larger than the area covered by one nanocavity and thus illuminates not only the NCs within the nanogap (70–100 per cavity), but also a large number of NCs that do not contribute to the hybridization (see Supplementarys Note 3 and 6 for (i) the local electric field distribution in the area of a nanocavity; (ii) the estimation of the mode volume and THz vacuum electric field; and (iii) the procedure for the visual quantification of the number of NCs occupying a nanocavity). This suggests that the Raman signature of the hybridized FR resonance within the cavity is enhanced by about 2 orders of magnitude (see Supplementary Note 5 and Supplementary Table 2 for calculation details), which is consistent with what  has recently been observed in the case of molecular bonds under strong vibrational coupling conditions^13^. It is important to note that this enhancement, unlike the traditional surface-enhanced Raman scattering mechanism^28^, affects only the hybridized phonon resonance and leaves the rest of the Raman spectrum unaltered.

### Tuning the plasmon resonance across the FR phonon mode

**Fig. 2:**
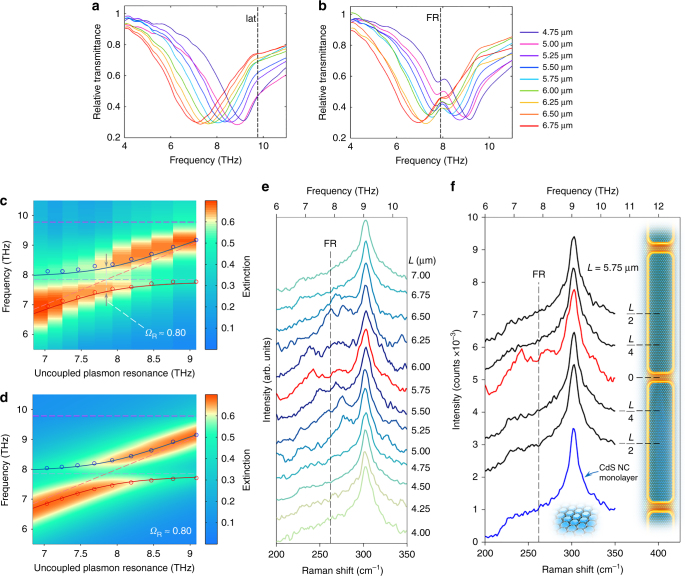
Polariton anti-crossing and Raman characterization. **a** Transmission response of bare arrays featuring different nanoantenna lengths. The vertical dashed line (labelled “lat”) marks the position of the lattice mode at around 9.8 THz (see below and Supplementary Note 6 for further details). **b** Same as in (**a**), when the arrays are covered with a single layer of NCs (dashed line: FR phonon resonance). **c**, **d** Experimental (**c**) and numerical (**d**) 2D maps of the polariton branches dispersion. The colour bar corresponds to the values of the extinction *E*, extracted from the transmission *T* as *E* = 1−*T*. The blue (red) solid lines are the trends of the high-energy (low-energy) polariton branch, as estimated by a three-coupled-oscillator model (see below and Supplementary Note 7), while the purple dashed line marks the lattice mode position. **e** Raman spectra of the NCs taken in a nanocavity region for different values of *L*. **f** Raman spectra of the NCs taken: in different positions along the nanoantennas composing a nanocavity (black lines), in the nanocavity region (red line) and just outside the cavity on the silicon substrate (blue line). The spectra are vertically shifted for clarity

A distinctive feature of strong coupling is the so-called “anti-crossing” behaviour^9,27^, which is connected with the creation of two separate polariton branches that do not intersect when the resonance of the optical cavity is tuned across the absorption band of the matter system. To examine anti-crossing in the context of our investigation, we prepared a series of plasmonic arrays featuring different nanoantenna lengths *L*, in such a way that their resonances spanned a wide THz frequency band comprising the FR phonon line (Fig. 2a). When covered with a monolayer of NCs, the transmission of these arrays (Fig. 2b) allows reconstructing the dispersion of the two polariton branches, as it is clearly visible in the colour map of Fig. 2c. The same behaviour is also well reproduced by the results of numerical simulations in Fig. 2d (see Supplementary Note 6 for simulation details). The Rabi splitting of the hybridized resonance (i.e., the separation of the two polariton branches when the uncoupled plasmon resonance is aligned to the uncoupled FR mode), as extracted from direct THz measurements, is estimated to be 0.8 THz. A thorough micro-Raman characterization of this series of arrays gives valuable information regarding the nanoscale response of the investigated plasmon–phonon strong coupling. As can be seen in Fig. 2e, f, the Raman spectrum is modified only within the nanocavity areas, and only when *L* is tuned to approximately match the plasmon resonance of the nanoantennas to the FR phonon mode, thus allowing the hybridization to occur. For *L* values corresponding to resonances well detuned from the FR line, as well as far from the nanocavities, either along the nanoantennas or away from the metal over the silicon substrate, the Raman spectrum of the NCs results to be not affected by the plasmonic structures. These evidences represent a clear proof of a nanoscale hybridization of the NC phonon response.

### Scaling of the Rabi splitting with the number of NC layers

**Fig. 3:**
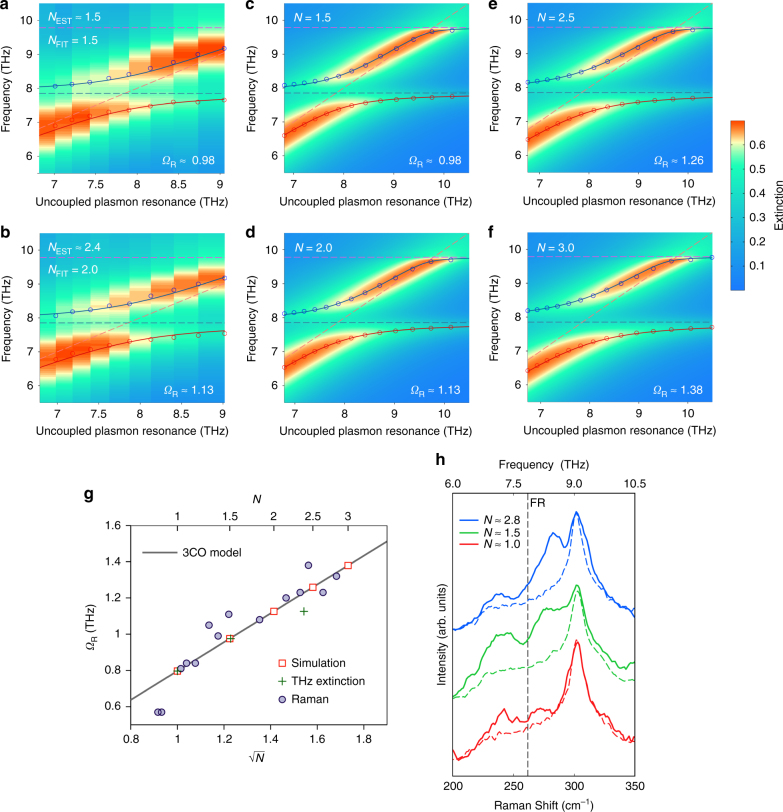
Rabi splitting vs. number of NC layers. **a**–**f** Experimental (**a**,** b**) and numerical (**c**–**f**) 2D maps of the polariton branches dispersion for different values of NC layer number *N*. As a reference for the experimental data, *N*_EST_ = 1 corresponds to an average number of NCs inside a nanocavity of around 86 (see Supplementary Note 3). The red (low-energy polariton) and blue (high-energy polariton) lines are the best fit with the three-coupled-oscillator (3CO) model. **g** Rabi splitting as a function of √*N*, extracted from: the three-coupled-oscillator model (black line), numerical simulations (red bordered squares), THz extinction (green crosses) and Raman measurements (purple circles). For each Raman data point, *N* is evaluated considering the number of NCs contained inside the specific nanocavity under measurement (see Supplementary Note 3). **h** Raman spectra for *L* = 5.75 μm and three different values of *N*. Solid lines indicate spectra taken in a nanocavity region and dashed lines indicate spectra taken just outside the respective cavity (using the same experimental parameters) on the silicon substrate

Finally, we have also explored how the plasmon–phonon strong coupling is affected by the number of NCs interacting with the plasmonic nanostructures. To do so, we followed a specific protocol (as detailed in the Supplementary Note 2) to add additional NCs over the previously formed layer. Figure 3a, b shows that the increase of the number of NCs over the surface results in a further splitting of the polariton branches, as indeed expected for strongly coupled systems^9^. Interestingly, however, the high-energy polariton branch in our measurements shows a peculiar dispersion at high frequencies and tends to intersect the uncoupled plasmon resonance line above 9 THz. We found that this is due to the presence in our system of a third characteristic resonance, namely a lattice mode^29^ at *v*_lat _≈ 9.8 THz (see Fig. 2a, as well as purple dashed lines in Fig. 2c, d and Fig. 3a–f). Lattice modes in plasmonic arrays are also known to effectively couple to other resonances^29,30^. Our overall system is thus better described by a three-oscillator model^31^ (i.e., involving plasmon, phonon and lattice resonances, see Supplementary Note 7), which well reproduces the observed pinning of the high-energy polariton branch for high frequencies, as shown by the fitting curves (solid lines) in Fig. 3a, b. This behaviour is also confirmed by numerical simulations, as it is shown in Fig. 3c–f for the case of *N* = 1.5, 2, 2.5 and 3 NC layers, respectively.

Even in the presence of the additional lattice resonance, the Rabi splitting *Ω*_R_ in our system is found to grow as √*N* (as shown by both the model—solid black line—and simulation results—red bordered squares—in Fig. 3g), which is the typical trend for two strongly coupled oscillators^9^. By exploiting this dependence in the fitting model, we can extract an estimate of the average increase of the number of NCs over the array surface (expressed as a fractional increase of the number of layers) for the measurements presented in Fig. 3a, b, which returns the values *N*_FIT_ = 1.5 and 2, respectively. This fit is found to be in fair agreement with the average number of layers we estimated (*N*_EST_ ≈ 1.5 and 2.4) by directly observing the SEM images of some tens of nanocavities (see Supplementary Note 3 and Supplementary Table 1). Figure 3g shows as well that the Rabi splitting observed in micro-Raman measurements (for nanocavities featuring different numbers of NCs, purple circles) also follows the theoretically predicted power-law dependence. Examples of these Raman spectra for three values of *N* are given in Fig. 3h, which furthermore highlights the significant modification of the Raman response induced by the additional NCs in a nanocavity region.

## Discussion

We have shown that the dipole-active phonon resonance of polar NCs can be hybridized by the strongly concentrated THz vacuum field of a plasmonic nanoantenna cavity. Evidence of strong plasmon–phonon coupling can be observed in the far field, by direct THz illumination of an extended array of nanoantennas covered with the NCs. A micro-Raman characterization of the surface in “dark” conditions (i.e., with no THz illumination) shows that the hybridization occurs just in the nanocavity regions, confirming the nanoscale nature of the phonon resonance reshaping. Vacuum Rabi splittings exceeding 15% of the uncoupled phonon resonance frequency are found in Raman spectra of cavities containing less than three NC layers, and are accompanied by a Raman signal enhancement >100. On the one hand, our findings open novel venues for engineering the optical phonon response of nanomaterials, which can have a significant impact for example in their light-emitting properties or in their electronic transport characteristics, due to the crucial role played by electron–phonon interactions in such systems^32–35^. On the other hand, nanoscale plasmon–phonon strong coupling can represent an innovative platform for THz science, for the exploration of enhanced and localized nonlinear phenomena as well as for the generation of coherent radiation in this still hardly accessible region of the electromagnetic spectrum.

### Data availability

The data that support the findings of this study are available from the corresponding authors upon reasonable request.

## Electronic supplementary material

Supplementary Information
