# Supplementary material for: Reshaping the phonon energy landscape of nanocrystals inside a terahertz plasmonic nanocavity
Source: Nat Commun. 2018 Feb 22;9:763. doi: 10.1038/s41467-018-03120-3 (PMC5823850; doi:10.1038/s41467-018-03120-3)
Supplement: Supplementary file 1 — Supplementary Information [file 41467_2018_3120_MOESM1_ESM.pdf]

Supplementary Information

Reshaping the Phonon Energy Landscape of Nanocrystals Inside a Terahertz Plasmonic Nanocavity

Xin Jin et al.

Supplementary Note 1: Fabrication of THz gold nanoantenna arrays

Matrices of nanoantenna chains were fabricated by means of the electron beam lithography (EBL) technique. After substrate cleaning in an ultrasonic bath of acetone, a 160-nm thick poly(methyl methacrylate) (PMMA) layer was spin-coated (1800 rpm) on a 500-μm thick, high resistivity (> 10 kΩ·cm) (100)-oriented silicon chip. Subsequently, annealing was performed at 180°C for 7 min. In order to prevent charging effects during electron beam exposure, a 10-nm thick Al layer was thermally evaporated on the PMMA surface. Electron beam direct-writing of the nanoantenna arrays was carried out using an ultra-high resolution Raith 150-Two e-beam lithography and imaging system. During exposure of the nanoantennas, a beam energy of 20 keV was employed, setting the exposure dose to 550 μC·cm^{-2}. After the Al conductive layer removal in a 1 M KOH solution, the exposed resist was developed in a solution of methyl isobutyl ketone (MIBK)/isopropanol (IPA) (1:3) for 30 s. Electron beam evaporation in a high vacuum chamber (base pressure 10^{-7} mbar) was exploited to produce a 5-nm adhesion layer of titanium and a 50-nm Au film, with a 0.3 Å·s^{-1} deposition rate. Finally, the unexposed resist was removed through a conventional lift-off process in hot acetone. To improve the lift-off efficiency, sonication for 2 minutes at 40 kHz frequency was employed. The substrate was then rinsed out in IPA for 30 s and dried in nitrogen. O_2 plasma ashing at 150 W for 10 minutes was used to remove residual PMMA resist and other organic residues. The fabricated two-dimensional arrays cover an area of 200 × 200 μm^2 and are composed of chains of 200-nm wide dipole antennas coupled end-to-end through a fixed gap G_x = 30 nm along their long-axis direction and featuring a spacing G_y = 8.5 μm along their short-axis direction.

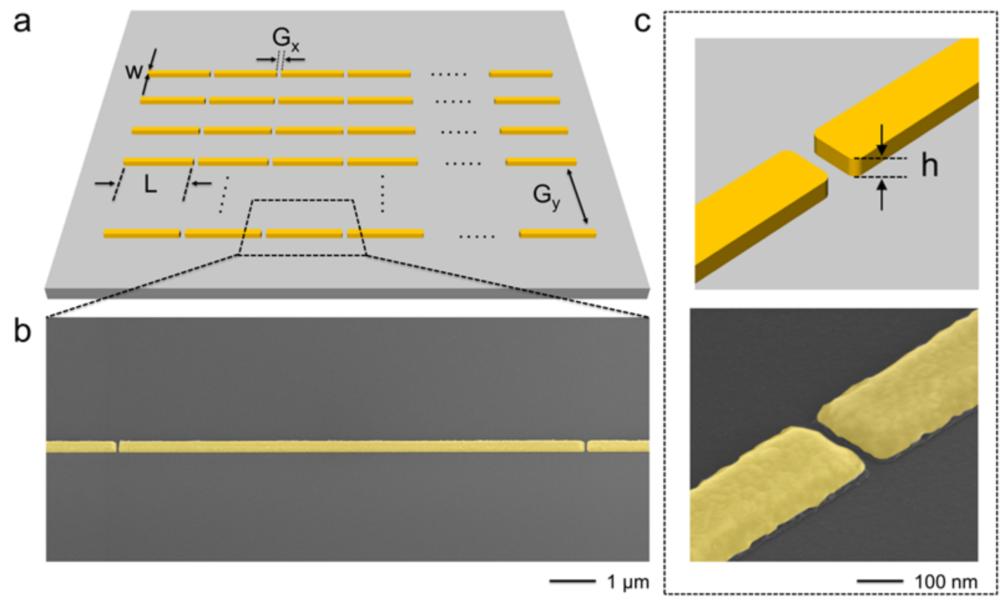

Supplementary Figure 1: **a,** Schematic view of THz antennas on the Si substrate. **b,** SEM image of an antenna stripe. **c,** Sketch showing the details of the cavity gap between two antennas (upper panel) and corresponding SEM picture in false colours (lower panel).

A schematic view of a THz nanoantenna array, together with two representative scanning electron microscope (SEM) images, is presented in Supplementary Figure 1. Arrays with different nanoantenna length L, ranging from 4 to 7 µm, were fabricated on the same silicon substrate in order to tune their plasmonic resonance in the THz frequency band comprising the Fröhlich (FR) optical phonon mode of the nanocrystals (NCs).

Supplementary Note 2: Synthesis and characterization of CdS NCs

Materials

Trioctylphosphine oxide (TOPO, 99%), trioctylphosphine (TOP, 97%), cadmium oxide (CdO, 99.999%), and sulfur powder (S, 99%) were purchased from Strem Chemicals. Octadecylphosphonic acid (ODPA, 99%) was purchased from PCI Synthesis. Octadecene (ODE, 90%), oleic acid (OA, 90%), toluene (99.7%), methanol (99.9%), 2-propanol (99.8%) and tetrabutylammonium iodide (TBAI, 99%) were purchased from Sigma Aldrich.

Methods

The NCs used in the present study were synthesized using a two-steps protocol, in which a CdS shell of desired thickness was grown on pre-synthesized CdS cores, following the procedure reported in Ref. [1] for CdSe/CdS giant-shell NCs.

Synthesis of CdS cores. CdS cores were obtained by mixing 3 g of TOPO, 0.280 g of ODPA and 0.060 g of CdO in a 25 mL tri-neck flask. The mixture was heated at 120°C under vacuum for 1 hour. Then, under nitrogen, the temperature was increased to 380°C to completely dissolve the CdO. When the CdO was dissolved and the solution became colourless, 1.5 mL of TOP was injected into the system and the temperature was allowed to recover to 380°C before the injection of TOP:S solution (0.020 g S+ 0.500 g TOP). The time of growth was set to 10 minutes to get cores of 6 nm in diameter. After the synthesis, the NCs were washed by precipitation and re-dissolution (3 times) in toluene and methanol. The final sample was dispersed in 3 mL of toluene.

Synthesis of CdS@CdS giant shell NCs. For the synthesis of core@shell CdS@CdS NCs, cadmium and sulfur precursors were prepared by respectively dissolving 0.640 g of CdO in 5 mL of OA and 0.160 g S powder in 5 mL of TOP. Both precursors were then diluted with ODE to get a 0.5 M Cd oleate solution in ODE (solution 1) and a 0.5 M TOP:S solution in ODE (solution 2).

In a 25 mL tri-neck flask a mixture of 3 mL of ODE and 1 mL of CdS cores solution was first degassed at 80°C under vacuum to remove water. Then, under nitrogen, the solution was heated to 300°C and a mixture of solution 1 and solution 2 (0.65 mL each) was injected into the system drop by drop in 1 hour, targeting a shell thickness of 2.35 nm, corresponding to the growth of 7 CdS monolayers (each layer having a thickness of 0.3358 nm, which is equivalent to half the lattice parameter along the crystallographic c axis).

The final sample was washed by centrifugation and re-dissolution with toluene and 2-propanol (3 times) and finally suspended in 3 mL of toluene.

Characterization of CdS cores and of CdS@CdS Giant Shell NCs

Transmission Electron Microscopy (TEM). Conventional TEM images were acquired on a JEOL JEM-1011 microscope equipped with a thermionic gun at 100 kV accelerating voltage.

Powder X-ray Diffraction (XRD) Analysis. XRD patterns were obtained using a PANalytical Empyrean X-ray diffractometer equipped with a 1.8 kW Cu Kα ceramic X-ray tube, PIXcel3D 2×2 area detector and operating at 45 kV and 40 mA. The diffraction patterns were collected in air at room temperature using a Parallel-Beam (PB) geometry and symmetric reflection mode. All XRD samples were prepared by drop casting a concentrated solution on a zero diffraction silicon wafer.

Inductively Coupled Plasma Optical Emission Spectroscopy (ICP-OES). ICP-OES analysis performed on an iCAP 6000 spectrometer (ThermoScientific) was used to estimate the concentrations of CdS cores and CdS@CdS Giant Shell NCs solutions. Prior to measurements, the samples were decomposed in aqua regia (HCl/HNO_3 = 3/1 (v/v)) overnight.

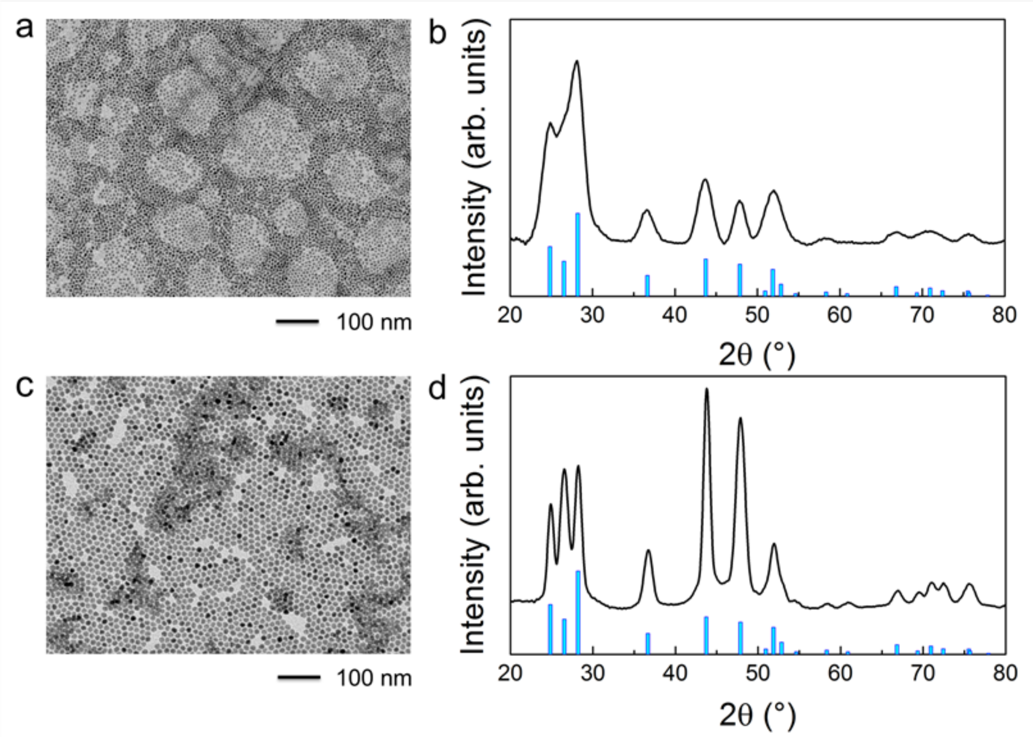

Supplementary Figure 2: **a,** TEM images and **b,** XRD patterns of CdS cores. **c,** TEM images and **d,** XRD patterns of CdS@CdS Giant Shell NCs.

Supplementary Figure 2 shows TEM and XRD results obtained for the CdS cores (panels a and b) and the CdS@CdS Giant Shell NCs (panels c and d). As evident from TEM characterizations, the two samples are quite monodisperse, with an average size of (5.6 ± 0.4) nm and (10.2 ± 0.6) nm for the CdS cores and the CdS@CdS Giant Shell NCs, respectively. XRD patterns confirm the crystallinity of both samples, showing an excellent match with reference card ICSD 98-015-4186, corresponding to the hexagonal wurtzite structure with $a = b = 4.1365$ Å and $c = 6.7160$ Å lattice parameters. Moreover, the absence of other peaks related to different phases and to precursors' residues certifies the high purity of the used samples. The concentration of the NCs solutions obtained following the previously described protocols was retrieved *via* ICP-OES. The CdS cores concentration was 34.24 μM, while the concentration of CdS@CdS Giant Shell NCs in the final sample solution was 10.95 μM.

Deposition of CdS@CdS Giant Shell NCs on THz gold nanoantenna arrays

The deposition of the CdS@CdS Giant Shell NCs on the THz gold nanoantenna arrays was carried out *via* spin coating under ambient conditions and at room temperature. Specifically, the solution obtained as described in the Methods subsection was diluted to a final NCs concentration of 1 μM. Then, 20 μL of the diluted solution were spin cast onto the patterned substrate at 2000 rpm for 60 s (acceleration of 1000 $rpm \cdot s^{-1}$). The procedure resulted in the deposition of a uniform monolayer of CdS@CdS Giant Shell NCs, as shown in Figure 1d of the main manuscript.

Thicker layers were obtained through multi-step layer-by-layer spin coating deposition; to avoid complete re-dissolution of the already deposited NCs, spin coating steps were interspersed with exposure of the sample to 20 μL of TBAI solution in methanol (10 $mg \cdot mL^{-1}$) for 30 s, followed by three rinse-spin steps with methanol, as described in Ref. [2].

Supplementary Note 3: Estimation of the number of NC layers

The estimation of the average number of NC layers over the sample surface was performed by directly counting in the SEM images the number of NCs within the nanocavities (where the THz electric field mainly concentrates over the sample surface, thus representing the key *loci* of the electromagnetic interaction) and averaging these values for around twenty-thirty nanogaps.

An example of this procedure, shown for a nanocavity covered with a single layer of NCs, is presented in Supplementary Figure 3.

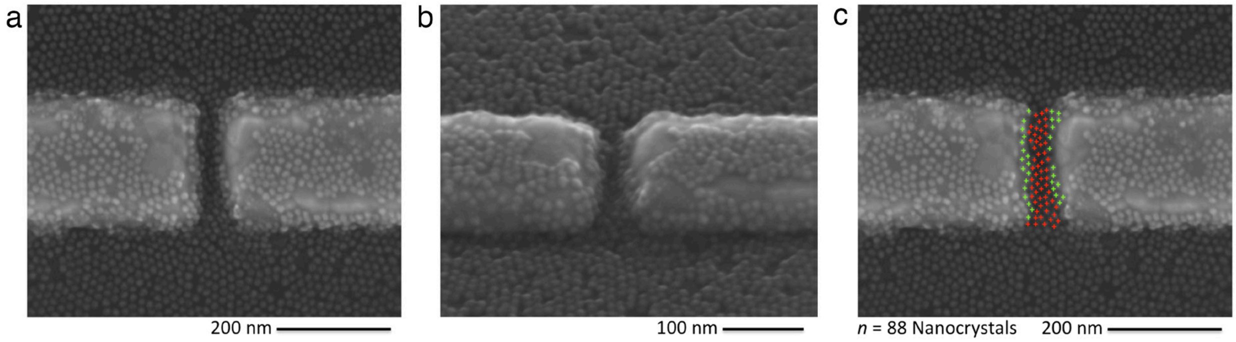

Supplementary Figure 3: Example of a visual estimate of the number of NCs in a plasmonic nanocavity; **a**, SEM top view. **b**, Tilted view, allowing a better evaluation of the NC positioning within the cavity. **c**, Actual counting over the top view (red crosses: NCs at the bottom of the cavity; green crosses: NCs on the cavity sidewalls).

For each nanocavity, several SEM images taken with different tilt angles were considered, in order to confirm the positioning of the NCs within the nanocavity and also evaluate their stacking in the case of multiple layers. From the average number of NCs obtained for the reference case of the monolayer n^1_{avg}, we then estimated the number of layers N_{EST} using the following formula $N_{EST} = n_{avg} / n^1_{avg}$, where n_{avg} is the average number of NCs extracted for the three different sample coverage conditions investigated in our work. The obtained values for N_{EST} are summarized in Supplementary Table 1 below.

To further corroborate these estimates, we also performed Electron Dispersive X-Ray (EDX) measurements, by employing a FEI Helios Nanolab 650 microscope working at an accelerating voltage $V = 5$ kV, current $I = 0.4$ nA and acquisition time $t = 120$ s. SEM images of the nanogaps were collected with 250.000× magnification, in order to include in the images the CdS NCs as well as the edges of the gold nanocavities. The relative atomic fractions of Cd and Au extracted from the EDX measurements taken in such a configuration were then used to estimate the equivalent number of NC layers, by fixing as a reference ($N_{EDX} = 1$) the case of a monolayer of NCs. As can be seen in Supplementary Table 1, the EDX estimate returns values for the effective number of layers that are in good agreement with the visual estimate described above.

Supplementary Table 1: Estimation of the NC layer number

N_{FIT}	n_{avg}	N_{EST}	N_{EDX}
**1.0**	86.4±8.7	1.00±0.14	1.00±0.14
**1.5**	130.1±11.4	1.51±0.20	1.49±0.26
**2**	205.7±10.4	2.38±0.28	2.21±0.29

As for the case of the Raman measurements, the number of layers N associated with each experimental data point refers to the actual number of NCs n contained in the specific nanogap under measurement, estimated by directly counting the NCs in the SEM image of that specific nanocavity ($N = n / n^1_{avg}$).

Supplementary Note 4: THz characterization

THz transmission spectra were collected at the high-brightness synchrotron infrared source SISSI@Elettra. THz radiation is generated at the bending magnet 9.1 and propagated in ultra-high-vacuum to the SISSI laboratory, where the radiation enters a Bruker 70v FTIR spectrometer equipped with a Si beam-splitter, and is then coupled to a Hyperion infrared microscope. The microscope is provided with an external detector port allowing to install a He-cooled Si bolometer. Thanks to the high brightness of synchrotron light, this set-up allows performing polarized THz microscopy.

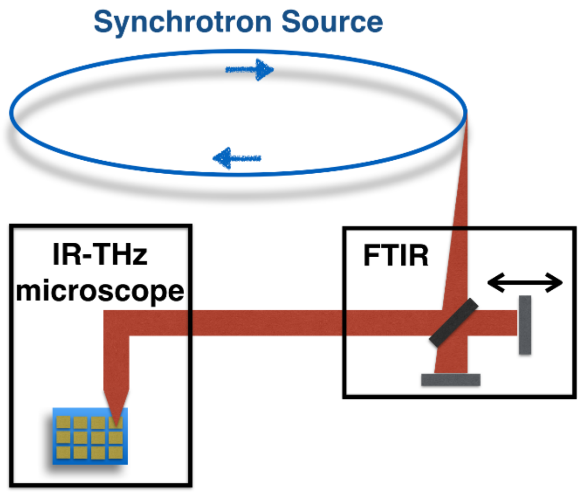

Supplementary Figure 4: Schematic layout of the experimental laboratory at the SISSI-mat beamline: a Bruker 70v FTIR spectrometer is directly coupled to the synchrotron source, and then a Hyperion infrared microscope is used to perform THz transmission measurements.

A grating polyethylene polarizer was positioned at the entrance of the microscope, oriented along the main linear polarization axis of synchrotron radiation, corresponding to the electron's orbit plane. Polarized transmittance measurements were performed by orienting the sample mounted on a goniometer, in such a way that the long axis of the nanoantennas was set parallel to the main polarization axis of the synchrotron beam. A Cassegrain objective (15×, mean incidence angle: 18°) was used to focus THz light onto the sample, while an aperture was employed to limit the focal spot size to a diameter of approximately 260 μm. Data were acquired by co-adding 512 scans with a spectral resolution of 4 cm^{-1} (spectral point spacing of 2 cm^{-1}).

Supplementary Note 5: Raman characterization

Experimental details of Raman measurements

Raman investigation was performed in a backscattering configuration using a micro-spectrometer system (Renishaw inVia) equipped with a 150× LEICA PL APO objective (numerical aperture NA = 0.95) and a thermo-electrically cooled CCD as detector (working temperature -60°C). Spectra were collected exciting the system at λ = 632.8 nm with a He:Ne laser; the wavelength was chosen after optimizing the experimental conditions in terms of scattering efficiency, suppression of fluorescence emission from the CdS NCs (bandgap ~2.4 eV), and beam size at the diffraction limit. The laser power was fixed at 1.7 mW with an integration time of 10 sec. The scans along the nanoantennas were performed using an XYZ motorized stage with a nominal 0.01 μm step. The polarization of the exciting laser source was set by means of a half waveplate.

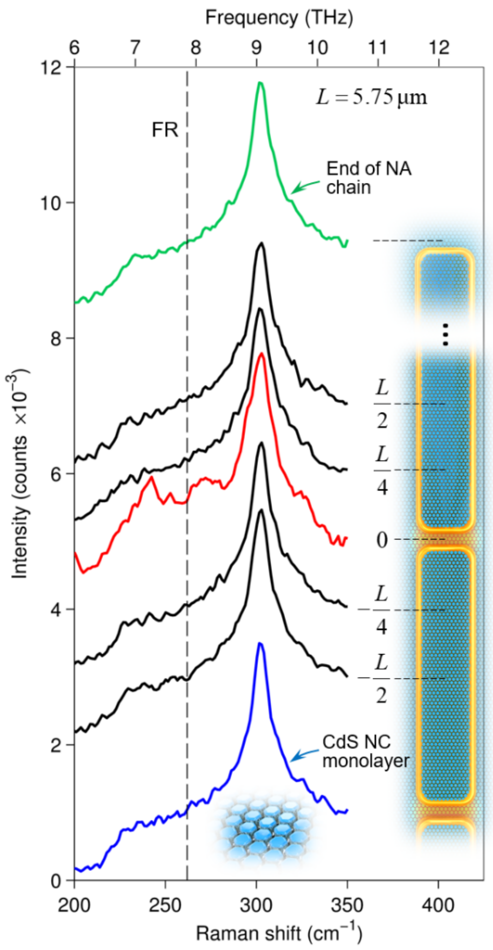

Supplementary Figure 5: Raman spectra taken in different positions on the nanoantenna array with L = 5.75 μm.

Supplementary Figure 5 shows some examples of these measurements, taken in different positions over the sample surface (in the case of an array with antenna length tuned to achieve the strong coupling condition within the nanogaps – i.e., L = 5.75 μm – and covered with a single

layer of NCs). When compared with Figure 2f in the main text, Supplementary Figure 5 additionally evidences that no sign of hybridization of the FR phonon resonance can be observed at the extremity of a nanoantenna chain (green spectrum), thus confirming the fact that the observed hybridization occurs only within the antenna nanocavities.

Supplementary Figure 6 shows Raman spectra acquired on a resonant plasmonic nanocavity using different laser illumination powers (100% power level corresponds to 1.7 mW on the sample surface). As can be seen, apart from differences in the signal-to-noise ratio, the spectral positions of the two hybridized peaks do not show any power dependence. For power levels below 85%, the two hybridized peaks cannot be distinguished anymore in the Raman spectra.

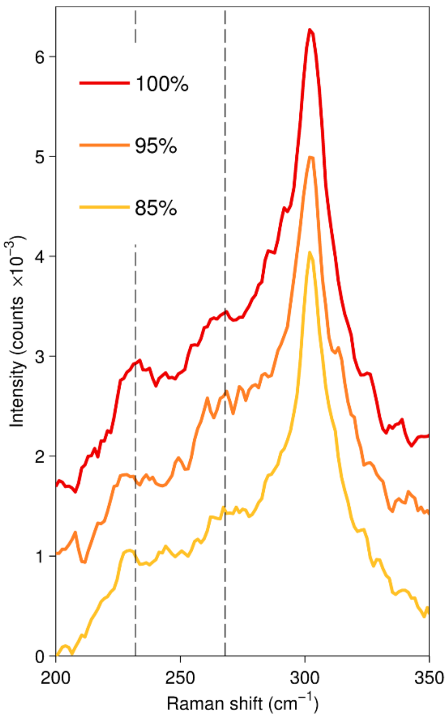

Supplementary Figure 6: Raman spectra taken at different laser power levels on a nanocavity featuring a number of NC layers $N \approx 1.5$.

Evaluation of the Rabi splitting from Raman spectra

Raman measurements were acquired illuminating a sampling area of ~1.39 μm^2. Rabi splitting values were calculated from the measured spectra using the following procedure.

A spectrum of interest (collected in a nanocavity region) was first corrected through linear baseline removal, and then by the subtraction of a reference spectrum (acquired on a spot just outside the cavity region). In this way, the contributions deriving from the transverse optical (TO), longitudinal optical (LO) and uncoupled FR phonon modes, as well as from the gold antenna luminescence background, were removed from the spectral data.

On the resulting signals (Supplementary Figure 7), a deconvolution in the region 200-300 cm^{-1} was operated, by fitting the peaks with two Lorentzian curves, thus extracting the hybridized resonance positions.

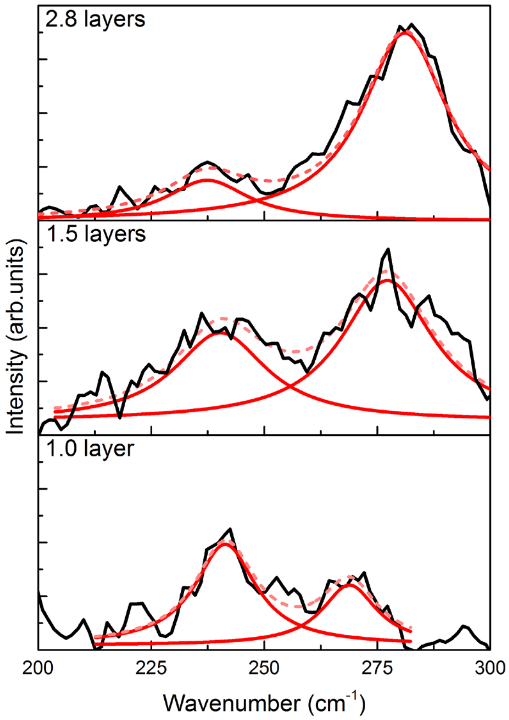

Supplementary Figure 7: Examples of the fitting procedure described above, for the Raman spectra reported in Figure 3h.

Polarization properties of Raman spectra

As can be seen in Figure 2f of the main text (by comparing the spectra taken on the nanoantennas with the one measured on the silicon substrate), no enhancement of the intensity of Raman scattering was observed for the "non-hybridized" phonon modes during the experiments. This behaviour can be explained considering that terahertz nanoantennas are far out of resonance for the exciting visible radiation (λ = 632.8 nm), thus not contributing to antenna-assisted SERS enhancement.

In order to further corroborate this result, additional Raman measurements were taken to compare spectra where the polarization of the exciting visible laser was set either parallel (red line in Supplementary Figure 8) or perpendicular (blue line) to the long axis of the antennas.

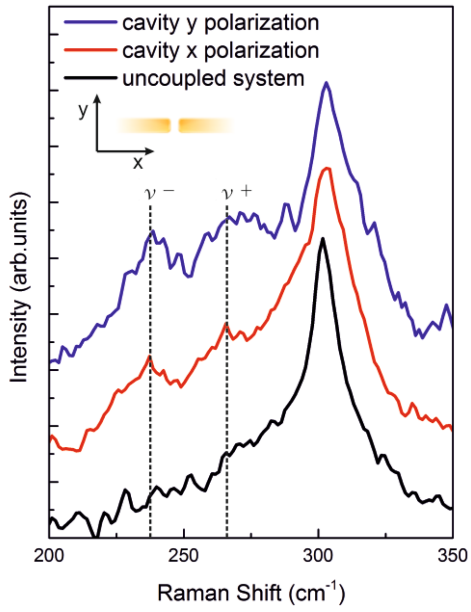

Supplementary Figure 8: Raman spectra collected in the same nanocavity with the polarization of the exciting laser source set parallel (red line) and perpendicular (blue line) to the long axis of the antennas. A spectrum collected just outside the gap region (black line) is also shown as reference.

As shown in Supplementary Figure 8, in both cases no sign of SERS enhancement was retrieved. Furthermore, no significant differences in the hybridized peak positions and amplitudes were found, confirming that the phonon hybridization observed in Raman measurements does not depend on the polarization properties of the exciting visible laser.

Besides this, additional Raman analysis was performed on resonant plasmonic nanocavities, by placing an output polarizer (analyser) in the path of the scattered light collected from the sample. As can be seen in Supplementary Figure 9, by increasing the angle formed by the main axis of the output analyser relative to the one of the input polarizer (the latter being set either along the long – Supplementary Figure 9a – or short – Supplementary Figure 9b – axis of the nanoantennas in these measurements) a progressive reduction of the intensity of the Raman response is observed for both the main LO peak and the hybridized FR peaks. All these peaks eventually disappear when the main axis of the output analyser is set perpendicular to the input polarization state. Indeed, the LO phonon resonance of CdS NCs is known to show a negligible degree of depolarization in Raman measurements, since the symmetric A1(LO) phonon mode is dominant [3]. These results thus seem to evidence, at least within our experimental configuration, a similar phonon symmetry between the hybridized FR response and the LO mode of the NCs.

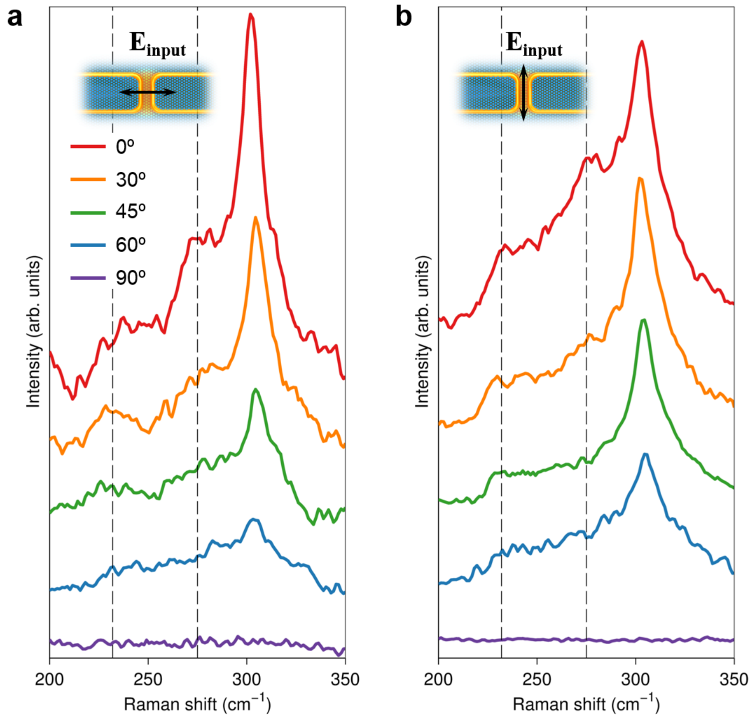

Supplementary Figure 9: Polarized Raman spectra taken with the input polarization set either along the long (**a**) or short (**b**) axis of the nanoantennas. The measurements in **a** and **b** were performed on two nanocavities with a similar number of NC layers $N \approx 2.3$.

Estimation of the Raman signal enhancement for the hybridized resonance

To extract an estimate of the enhancement related to the plasmon-phonon hybridization in our Raman measurements, we employed the following procedure:

We can consider the measured Raman signal $R_{\mathrm{MEAS}}(\nu)$ in a nanocavity region as the weighted average of the Raman response inside the nanocavity $R_{\mathrm{IN}}(\nu)$ and the one outside the nanocavity $R_{\mathrm{OUT}}(\nu)$, according to the following equation:

$$R_{\mathrm{MEAS}}(\nu) = \frac{B-A}{B} R_{\mathrm{OUT}}(\nu) + \frac{A}{B} R_{\mathrm{IN}}(\nu), \tag{1}$$

where $A = 0.006\ \mu\mathrm{m}^2$ is the area of a nanocavity and $B = 1.39\ \mu\mathrm{m}^2$ is the spot size of the micro-Raman excitation laser on the sample. Using Eq. (1), we can then estimate the Raman signal enhancement of the hybridized Raman peaks $K_{\nu\pm}$ as: $K_{\nu\pm} = \dfrac{R_{\mathrm{IN}}(\nu\pm)}{R_{\mathrm{OUT}}(\nu_{\mathrm{FR}})}$. The table below summarizes the enhancement values (spanning the range ~ 100 - 400), as extracted from the data reported in Figure 3h of the main manuscript.

Supplementary Table 2: Enhancement values for the hybridized Raman peaks in Figure 3h of the main text

N	$K_{\nu-}$	$K_{\nu+}$
**1.0**	185	90
**1.5**	262	380
**2.8**	101	384

Supplementary Note 6: Numerical simulation details

Material properties for the simulation model

Electromagnetic simulations were performed using a commercial Finite Element Method - based software: COMSOL Multiphysics, Optics Module. For the silicon substrate, we assumed a constant refractive index of 3.42. The complex permittivity of gold at THz frequencies was taken from Ref. [4]. To extract the dielectric properties of the layer of CdS NCs, we employed the following procedure: we prepared a thick layer (2.5 μm) of NCs over a silicon substrate and characterized its THz transmission (Supplementary Figure 10a, blue circles). We then fitted this measurement (red solid line), considering an effective permittivity $\varepsilon_{\mathrm{eff}}$ for the NC layer derived from the Maxwell-Garnett approximation for a dielectric mixture [5]:

$$\frac{\varepsilon_{\mathrm{eff}}-\varepsilon_{\mathrm{b}}}{\varepsilon_{\mathrm{eff}}+2\varepsilon_{\mathrm{b}}}=f\,\frac{\varepsilon_{\mathrm{CdS}}-\varepsilon_{\mathrm{b}}}{\varepsilon_{\mathrm{CdS}}+2\varepsilon_{\mathrm{b}}},\tag{2}$$

where ε_{b} and $\varepsilon_{\mathrm{CdS}}$ are the permittivities of the background (air in our case, $\varepsilon_{\mathrm{b}}=1$) and filling (CdS NCs) materials, respectively, while f is a dimensionless quantity representing the filling ratio of the mixture. For $\varepsilon_{\mathrm{CdS}}$, we considered the common formula used to describe the permittivity of a material in the reststrahlen region [6]:

$$\varepsilon_{\mathrm{CdS}}(\omega)=\varepsilon_{\infty}\left(1+\frac{\omega_{\mathrm{LO}}^{2}-\omega_{\mathrm{TO}}^{2}}{\omega_{\mathrm{TO}}^{2}-\omega^{2}-i\omega\gamma}\right),\tag{3}$$

where γ is a damping constant, ε_{∞} the high-frequency dielectric permittivity, and ω_{LO}, ω_{TO} the LO and TO phonon frequencies, respectively.

In the fitting procedure of the experimental data of Supplementary Figure 10a, we used the following literature values relative to bulk crystalline CdS: ε_{∞} = 5.3, ω_{TO} = 240 cm^{-1}, ω_{LO} = 302 cm^{-1} [7]. We then tuned the filling factor to match the experimental resonance position at around 7.9 THz, obtaining $f=0.75$, a value that is consistent with the densely

packed arrangement of our CdS layer. Finally, we tuned the damping constant to well reproduce the width of the resonance, which led to a value γ = 15 cm^{-1}.

The NC self-assembled layers under investigation were then simulated in our numerical analysis by employing an equivalent layer with effective permittivity as in Eq. (2) and appropriate thickness. This allowed us to drastically simplify the meshing requirements in the simulation domain and speed up the calculations. Supplementary Figure 10b shows the absorption of a single layer of NCs, obtained adopting this procedure.

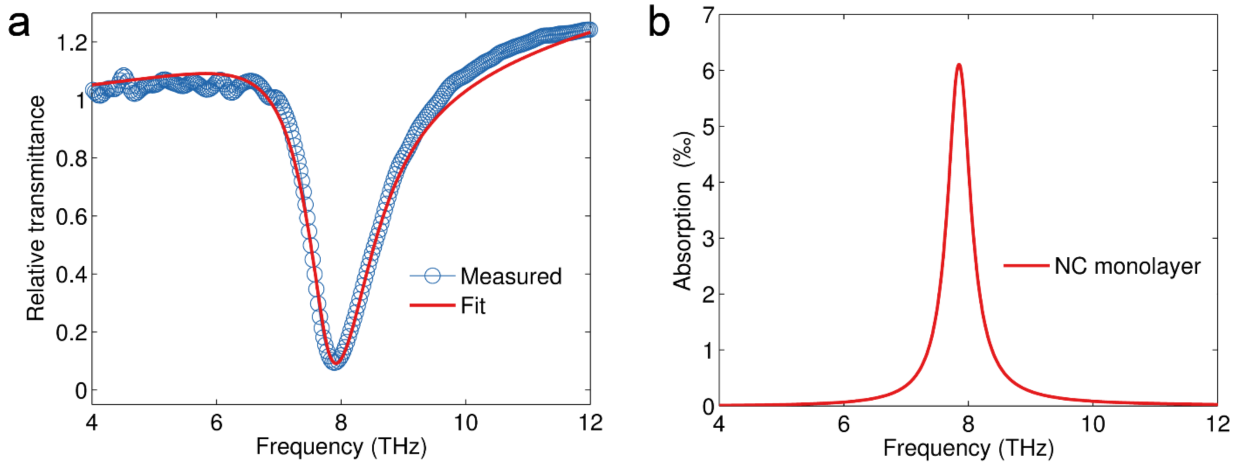

Supplementary Figure 10: **a,** Relative transmittance spectra (blue circles: experimental measurement, red line: fit with Eq. (2)) of a 2.5-µm-thick CdS NC layer. The relative transmittance is obtained normalizing the sample transmission with the one of a bare silicon substrate. **b,** Absorption spectrum of a monolayer of CdS NCs, as obtained using Eq. (2) and the parameters extracted from the fit in **a**.

Calculation domain and illumination conditions

In our simulations, we considered nanoantenna arrays on a silicon substrate with spacing G_x = 30 nm along the nanoantenna long axis (x) and G_y = 8.5 µm in the perpendicular direction (y). The nanoantenna width was set to 200 nm, the height to 55 nm, while the nanoantenna length was varied in the range 4 – 7 µm, to tune the array resonance peak position. All the nanoantenna sharp edges were rounded with a radius of curvature of 20 nm, except the ones perpendicular to the substrate in correspondence of the nanocavities, where a curvature of 40 nm was introduced to better resemble the fabricated structures.

The response of an infinite nanoantenna array was obtained by imposing appropriate periodic boundary conditions in the plane of the array. Two perfectly matched layers (PMLs) were instead set as boundary conditions at the top (air) and bottom (silicon substrate) of the calculation domain, to avoid spurious reflections.

For the input illumination conditions in the simulations, we employed a linearly polarized plane wave. The plane of incidence was tilted by 45° with respect to the nanoantenna long axis, while the angle of incidence in this plane was set to 18° (in accordance with the mean incidence angle of the Cassegrain objective, see Supplementary Note 4). The polarization state of the wave was then set taking into account the input polarization (parallel to the nanoantenna long axis)

impinging on the Cassegrain objective. We found that this type of illumination well reproduces the transmission response of the arrays under the Cassegrain objective as well as the experimental position of the lattice mode at around 9.8 THz (Supplementary Figure 11).

Finally, since in the experiments the size of the nanoantenna array (200 × 200 μm^2) is smaller than the estimated area of the illuminating THz spot (diameter $D \approx 260$ μm), the transmission results of the simulations (which assumed an infinite array) were normalized as follows, to be compared with the experimental results: $T'_{\mathrm{num}} = 0.76T_{\mathrm{num}} + 0.24T_{\mathrm{sub}}$. In such expression, T_{num} is the numerical transmission of an infinite nanoantenna array and T_{sub} is the transmission of a bare silicon substrate (the coefficients 0.76 and 0.24 take into account the area of the THz spot effectively covered by the array). The relative transmittance was then obtained in the same way as in the measurements: $T_{\mathrm{rel}} = T'_{\mathrm{num}} / T_{\mathrm{sub}}$.

Array response with and without CdS NCs

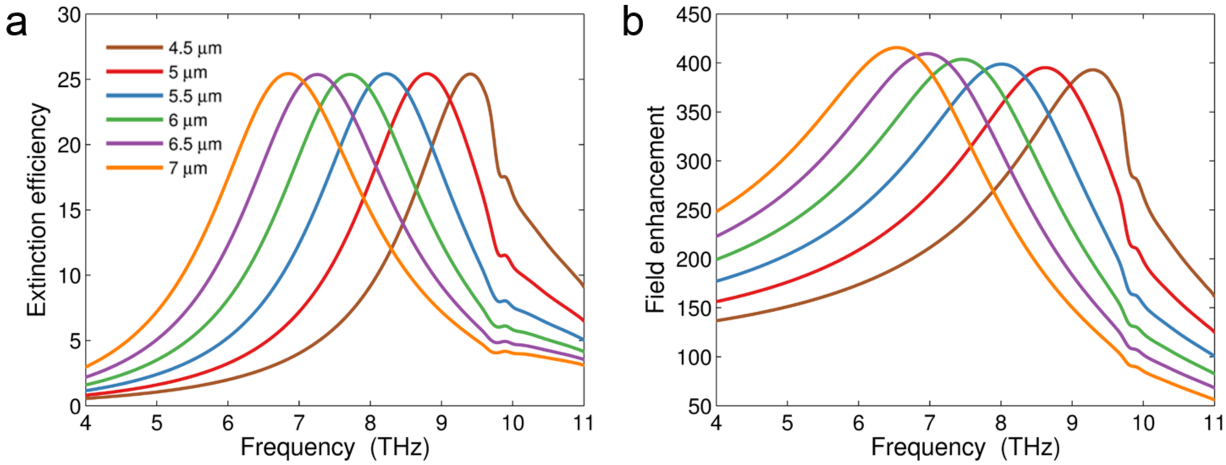

Supplementary Figure 11: **a,** Extinction efficiency for six different nanoantenna lengths. **b**, Corresponding field enhancement values, estimated in the centre of the nanocavity.

Supplementary Figure 11a shows the nanoantenna extinction efficiency (the ratio of the extinction cross section to the nanoantenna geometrical area) for arrays with different nanoantenna lengths as a function of frequency. Supplementary Figure 11b shows instead the dispersion of the near field enhancement value (the ratio of the local electric field to the background field) in the centre of the nanocavities. In both figures, the presence of a lattice mode at around 9.8 THz is clearly visible.

The lattice modes of a plasmonic array [8,9] arise from the mutual electromagnetic coupling of the individual plasmonic scatterers, associated with a grazing (diffracted) wave in the plane of the array. When a lattice mode is close to the plasmonic resonance of the array elements, the energy transfer between these two excitations can reshape the overall spectral response of the array, also leading to an increase of its resonance quality factor [10-12].

Lattice resonances are known to be found in correspondence of the so-called Wood-Rayleigh anomalies [8,9]. For our specific configuration, the lowest-energy anomaly appears at the frequency that satisfies the following equation:

$$k_{\text{sub}} = \left|\mathbf{k}_x + \mathbf{k}_y - \mathbf{A}_y\right|, \tag{4}$$

where $k_{\text{sub}} = k_0 n_{\text{sub}} = \frac{2\pi\nu n_{\text{sub}}}{c}$ is the absolute value of the wave vector in the array substrate, $\mathbf{k}_x = \hat{e}_x k_0 \sin\theta\cos\varphi$ and $\mathbf{k}_y = \hat{e}_y k_0 \sin\theta\sin\varphi$ are the x and y in-plane components of the impinging light wave vector (with φ the angle formed by the plane of incidence with the long axis of the nanoantennas and θ the angle of incidence within this plane), while $\mathbf{A}_y = \hat{e}_y \frac{2\pi}{G_y}$ is the reciprocal lattice vector in the y direction.

Supplementary Figure 12 shows how the lattice mode can be straightforwardly tuned by changing the spacing G_y of the array, and also evidences the agreement between the lattice resonance positions that can be estimated using Eq. (4) (vertical lines, evaluated using the parameters of our investigation, i.e. $n_{\text{sub}} = 3.42$, $\varphi = 45°$, $\theta = 18°$) and the ones visible in the electromagnetic simulations (coloured curves).

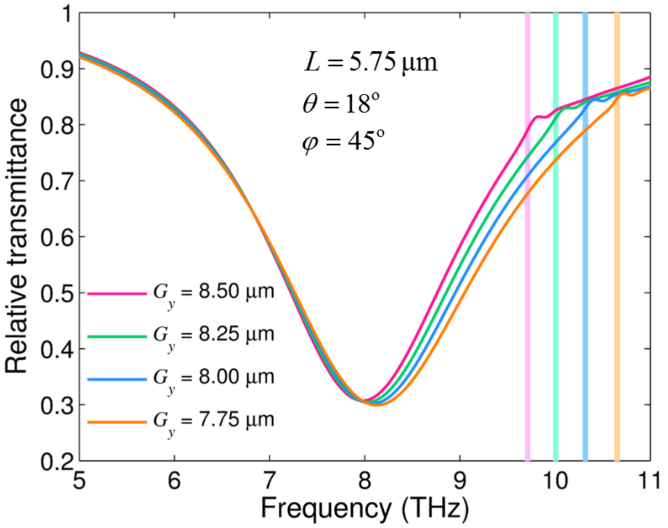

Supplementary Figure 12: Lattice mode dependence on the array spacing, estimated for a fixed antenna length of 5.75 µm. The coloured curves are the simulated transmission spectra, while the corresponding vertical lines indicate the lattice resonance positions calculated using Eq. (4).

The 2D maps of the extinction as a function of frequency and nanoantenna length are shown in Supplementary Figure 13 ($G_y = 8.5$ µm, corresponding to the case of the fabricated arrays) for bare arrays (a), and for arrays covered with a single "effective" layer of CdS NCs (b); effective layer thickness: 10 nm). A clear anti-crossing behaviour around the CdS phonon resonance is visible in Supplementary Figure 13b. The geometrical arrangement of the CdS layer in the simulations and the field distribution in the nanocavity region are shown in Supplementary Figure 13c,d, respectively.

Finally, the simulated transmission spectra of the bare nanoantenna arrays, as well as of the arrays covered with 1, 2, and 3 CdS NC layers are plotted in Supplementary Figure 14a, b, c, and d, respectively.

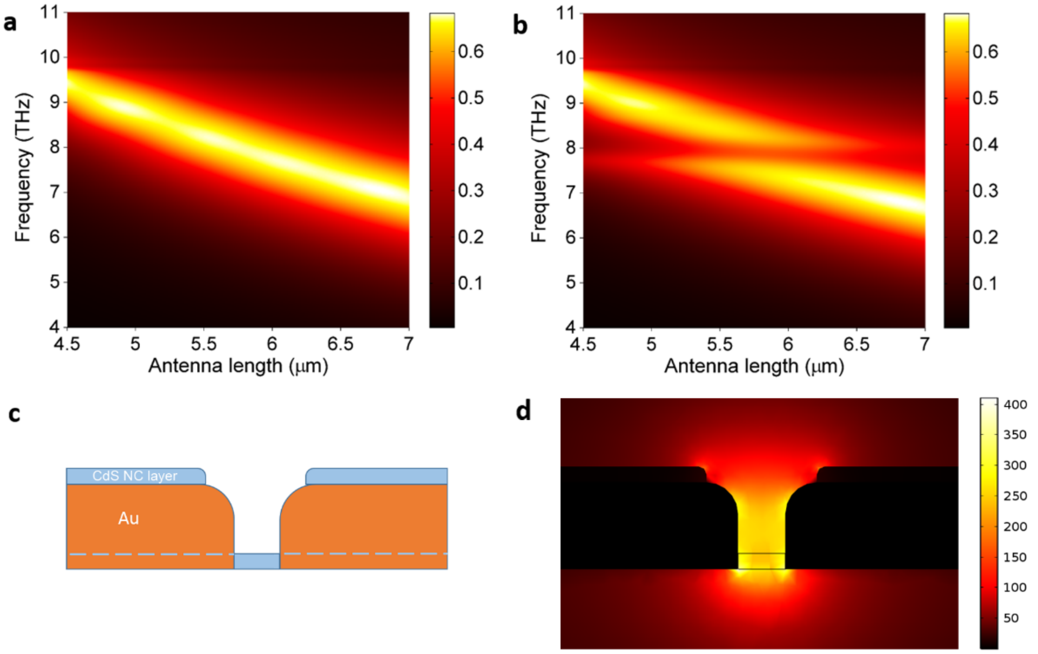

Supplementary Figure 13: **a,** 2D map showing the extinction spectra for different nanoantenna lengths, in the case of the bare arrays. **b,** same as in **a**, when the arrays are covered with a 10-nm-thick "effective" layer of CdS NCs. **c,** Lateral view of the geometrical arrangement adopted in the simulations for the CdS NC layer. **d,** Corresponding electric field distribution (frequency: 8 THz, nanoantenna length: 5.75 μm).

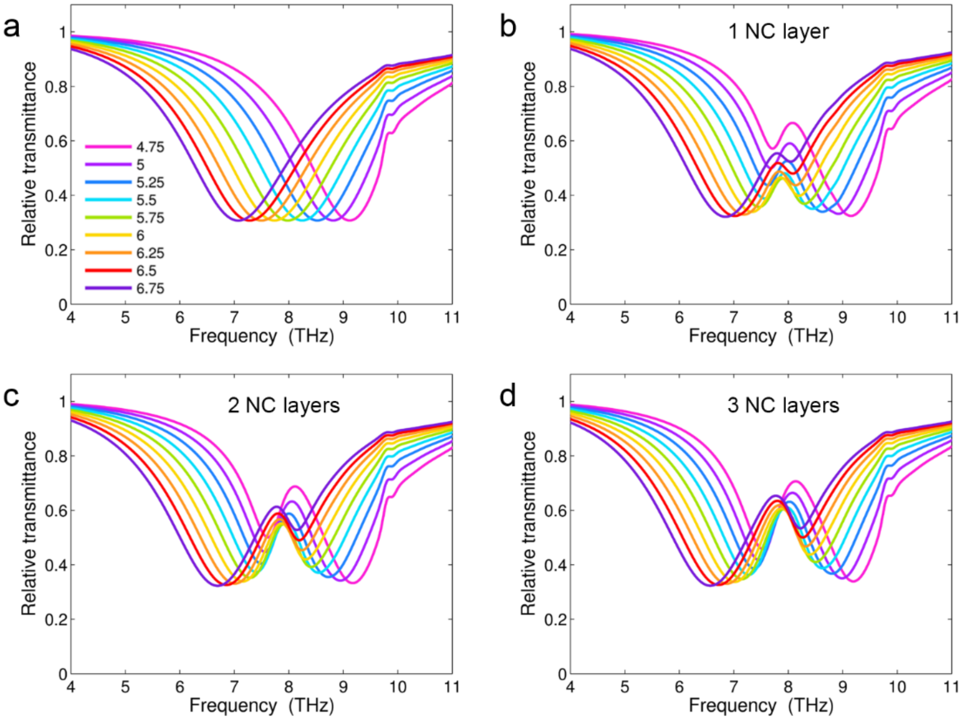

Supplementary Figure 14: Simulated transmission spectra for bare arrays (**a**), and arrays covered with 1 (**b**), 2 (**c**), and 3 (**d**) CdS NC layers.

Estimation of mode volume and THz vacuum electric field

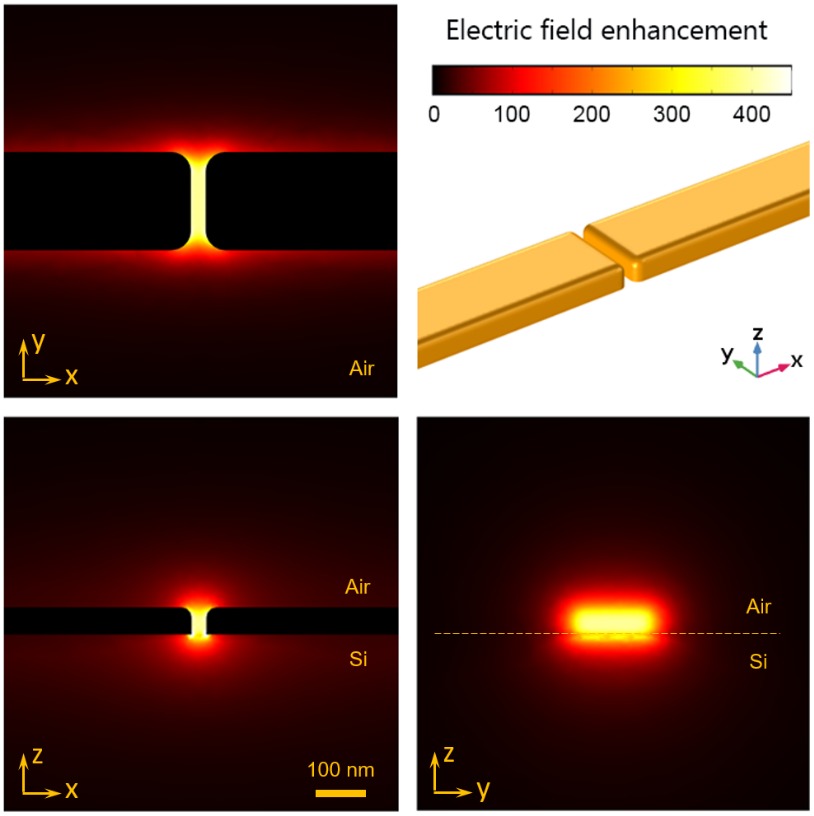

Supplementary Figure 15: The local electric field distribution in the area of a nanocavity. (all the 2D projections are taken on a plane passing through the nanocavity geometrical centre). Frequency: 8 THz; nanoantenna length: 5.75 μm

Supplementary Figure 15 shows the electric field distribution in the nanocavity area at resonance, on the planes perpendicular to the main simulation axes. Starting from the distribution of the local electric field in the 3D space, the mode volume of the plasmonic nanocavity can be estimated as follows [13]:

$$V_{\text{mod}} = \frac{\int \varepsilon(\mathbf{r})\left|E(\mathbf{r})\right|^2 d\mathbf{r}}{\max\left(\varepsilon(\mathbf{r})\left|E(\mathbf{r})\right|^2\right)}, \tag{5}$$

where the numerator can be read as the full domain integral of the energy density, and the denominator as the maximum value of the energy density extracted in the same domain. Considering the case of the array with nanoantenna length of 5.75 μm (corresponding to a resonance peak at around 8 THz, well aligned to the NC phonon resonance) we obtain a mode volume at resonance as small as $V_{\text{mod}} = 1.43 \times 10^6$ nm^3. Compared to the geometrical volume of the nanocavity ($V_{\text{geo}} \approx 200 \times 30 \times 55$ nm$^3 = 0.33 \times 10^6$ nm^3), the mode volume is thus just ~4.3 times larger. A graphical comparison of these two volumes is given in Supplementary Figure 16, in which the effective squeezing of the radiation in the nanocavity region becomes evident.

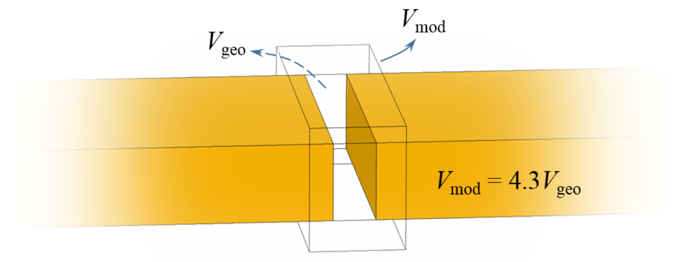

Supplementary Figure 16: Size comparison between the geometrical nanocavity volume and the mode volume.

Having an estimate of the mode volume, we can also evaluate the THz vacuum electric field within the cavity [14]:

$$|E_{\text{vac}}| = \sqrt{h\nu_{\text{res}} / \left(2\varepsilon\varepsilon_0 V_{\text{mod}}\right)}, \tag{6}$$

where ν_{res} is the resonance frequency, h the Planck's constant, ε and ε_0 the relative and vacuum permittivities, respectively. Using Eq. (6), we find $|E_{\text{vac}}| = 4.6\times10^5$ V·m^{-1} in air.

Supplementary Note 7: Three-coupled-oscillator model

As shown in the main text, due to the presence of the lattice mode at around 9.8 THz, our overall system in THz transmission measurements is better described by a three-coupled oscillator model [15,16]. The following matrix equation can be used to retrieve the characteristic frequency dispersion of the investigated interaction:

$$\begin{pmatrix} \nu_{\text{pl}} - \nu & V_{\text{pl-ph}} & V_{\text{pl-lat}} \\ V_{\text{pl-ph}} & \nu_{\text{ph}} - \nu & 0 \\ V_{\text{pl-lat}} & 0 & \nu_{\text{lat}} - \nu \end{pmatrix} \begin{pmatrix} \alpha \\ \beta \\ \gamma \end{pmatrix} = 0, \tag{7}$$

where ν_{pl}, ν_{ph}, and ν_{lat} are the frequencies of the noninteracting plasmon, phonon, and lattice modes, respectively, while ν are the eigenfrequencies of the coupled system. $V_{\text{pl-ph}}$ ($V_{\text{pl-lat}}$) is the coupling constant between the plasmon and the phonon (lattice) modes. Finally, α, β and γ represent the coefficients of the basis functions of the bare plasmon, phonon and lattice modes, respectively. By diagonalizing this matrix, the dispersion of the hybrid eigenfrequencies can be readily evaluated. While this model does not include damping, it is worth underlining that such an idealized picture has proven to be successful in describing experimental systems with finite linewidths, as shown in a variety of studies, for both two- [17,18] and three- [15,16] coupled-oscillator interactions. From an operative point of view, a real system with finite resonance linewidths can be considered as strongly coupled whenever the two hybridized resonances are clearly visible in the response spectrum (see, for example, [19,20]). This is evidently the case of our plasmon-phonon interaction, since the double peak formation can be distinctly seen not only in the direct THz extinction response, but also in the Raman measurements, with no THz illumination. Such splitting is indeed an intrinsic characteristic of the coupling between the

matter transition and the cavity vacuum-field, and thus it manifests itself in any optical property of the system.

In order to compare the above model to our numerical and experimental results, the uncoupled plasmon resonance frequency needs to be estimated. The resonance wavelength λ_{Res} of nanorod-shaped plasmonic nanoantennas typically obeys the following relation: $\lambda_{\mathrm{Res}} \approx 2n_{\mathrm{eff}}L$ [21,22], where n_{eff} is the effective index of the nanorod surface mode. While this equation well describes the behaviour of an isolated nanoantenna in the THz range, an additional parameter needs to be added in the case of an array of interacting nanoantennas: $\lambda_{\mathrm{Res}} = 2n_{\mathrm{eff}}L + S$, to also take into account the expected red-shift of the resonance induced by the near-field coupling between the nanostructures [23]. In particular, by fitting the resonance wavelength dependence on the nanoantenna length for the case of bare nanoantenna arrays with the above formula (far from the lattice mode), we then obtain $n_{\mathrm{eff}}^{0} = 2.375$ and $S = 10.2$ μm (see red circles and related fit in Supplementary Figure 17). The uncoupled plasmon resonance frequency in our system can be thus written as:

$$\nu_{\mathrm{pl}} = \frac{c}{2n_{\mathrm{eff}}L + S}, \tag{8}$$

with c being the speed of light in vacuum.

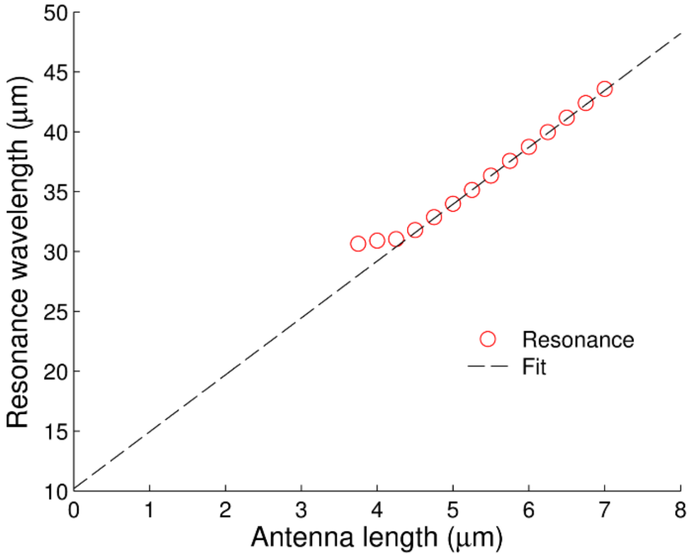

Supplementary Figure 17: Resonance wavelength as a function of the antenna length for bare arrays.

The above relation (Eq. (8)) has been used to fit the numerical and experimental polariton dispersion curves with the model expressed in Eq. (7). Supplementary Figure 18 shows the result of this procedure on the numerical data regarding a monolayer of NCs.

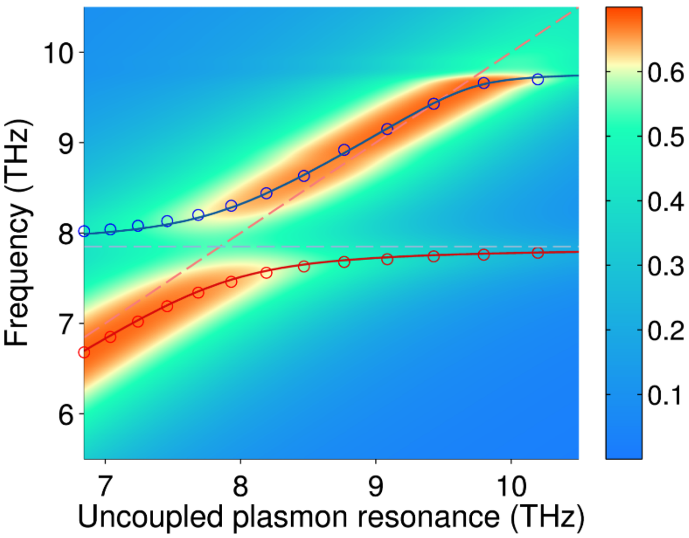

Supplementary Figure 18: Polariton trace fit of the simulation results for the case of 1 NC layer (compared to Figure 2d in the main text, here the axes are extended to frequency values above 10 THz).

From these fits, we can also extract the quantities $|\alpha|^2, |\beta|^2$ and $|\gamma|^2$, which can be used to estimate the relative contribution of the plasmon, phonon, and lattice modes to the overall hybridized state. As can be seen from Supplementary Figure 19 below, at the FR resonance frequency an essentially complete hybridization of the plasmon-phonon modes occurs (i.e., $|\alpha|^2$, $|\beta|^2 \approx 0.5$), while the contribution of the lattice mode is negligible.

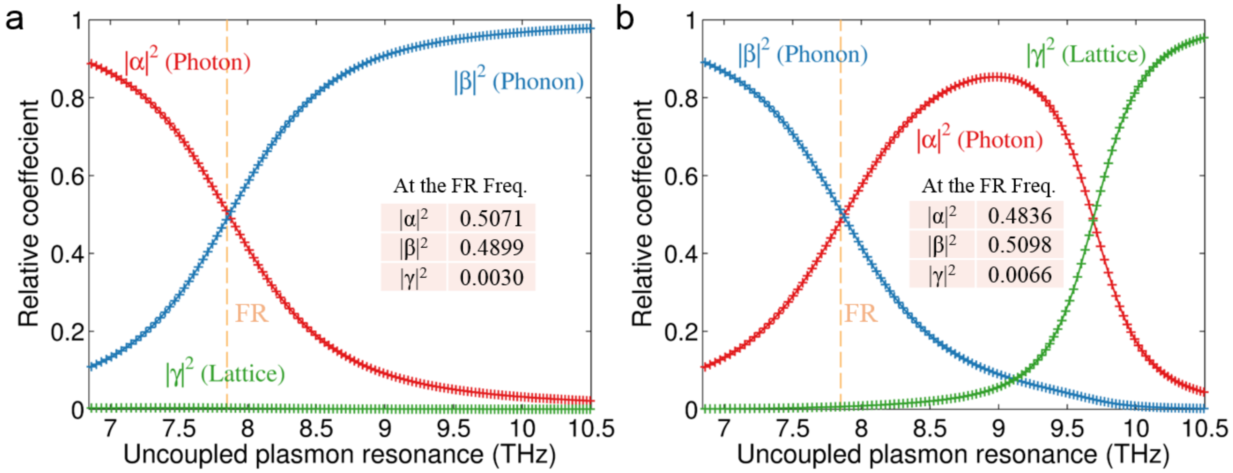

Supplementary Figure 19: **a**, Relative coefficients for the low-energy polariton branch, in the case of 1 NC layer. **b**, Same as in **a**, for the high-energy polariton branch.

Supplementary Table 3 (for the simulations) and Supplementary Table 4 (for the experiments) below summarize all the parameters used in the fitting procedure and also include the root-mean-square error (RMSE) analysis for the two polariton branches: $RMSE = \sqrt{\frac{1}{m}\sum_{j=1}^{m}\left(y_j - \hat{y}_j\right)^2}$ where y_j are the analytical model estimates for the polariton branch

positions and $\hat{y}_j$ the m experimental or simulation points. As can be seen, in both simulations and experiments, and for the various numbers of NC layers considered in this study, the following parameters were kept as constant in the fitting procedure: $\nu_{\text{ph}} = 7.85$ THz, $\nu_{\text{lat}} = 9.78$ THz, $S = 10.2\,\mu\text{m}$, $V_{\text{pl-lat}} = 0.18$ THz. The plasmon-phonon coupling constant was considered to obey the relation [24]: $V_{\text{pl-ph}} = V^1_{\text{pl-ph}}\sqrt{N}$, where $V^1_{\text{pl-ph}}$ is the value of this coupling parameter for 1 layer of NCs and N is the number of layers. Interestingly, we found that for the best fit of both the experimental and numerical data, the effective index n_{eff} needs to be slightly increased when adding the NC layers, according to the empirical rule: $n_{\text{eff}} = \gamma_{\text{eff}} N + n^0_{\text{eff}}$, with $\gamma_{\text{eff}} = 0.025$. This behaviour is consistent with the increase in the average permittivity around the nanoantennas induced by the presence of the NC layers. For both the experimental and simulation polariton traces, the RMSE values associated with the fitting curves are of the order of some tens of GHz, well below the Rabi splitting values (≥ 800 GHz) extracted from our THz response data.

Supplementary Table 3: Fitting coefficients for the simulated polariton traces

	1 layer	1.5 layers	2 layers	2.5 layers	3 layers	Units
$V_{\text{pl-ph}}$	0.40	0.49	0.57	0.63	0.69	THz
$V_{\text{pl-lat}}$	0.18	0.18	0.18	0.18	0.18	THz
ν_{ph}	7.85	7.85	7.85	7.85	7.85	THz
ν_{lat}	9.78	9.78	9.78	9.78	9.78	THz
n_{eff}	2.400	2.413	2.425	2.438	2.450	/
S	10.2	10.2	10.2	10.2	10.2	µm
$RMSE\ (\nu_+)$	0.025	0.025	0.019	0.020	0.024	THz
$RMSE\ (\nu_-)$	0.016	0.009	0.004	0.009	0.018	THz

Supplementary Table 4: Fitting coefficients for the experimental polariton traces

	1 layer	~1.5 layers	~2 layers	Units
$V_{\text{pl-ph}}$	0.40	0.49	0.57	THz
$V_{\text{pl-lat}}$	0.18	0.18	0.18	THz
ν_{ph}	7.85	7.85	7.85	THz
ν_{lat}	9.78	9.78	9.78	THz
n_{eff}	2.400	2.413	2.425	/
S	10.2	10.2	10.2	µm
$RMSE\ (\nu_+)$	0.085	0.035	0.053	THz
$RMSE\ (\nu_-)$	0.065	0.049	0.069	THz

Supplementary References

[1] Christodoulou, S. *et al.* Synthesis of highly luminescent wurtzite CdSe/CdS giant-shell NCs using a fast continuous injection route. *J. Mater. Chem. C* **2**, 3439-3447 (2014).

[2] Chuang, C.-H. M., Brown, P. R., Bulović, V. & Bawendi, M. G. Improved performance and stability in quantum dot solar cells through band alignment engineering. *Nat. Mater.* **13**, 796-801 (2014).

[3] Del Gobbo, S. Cadmium sulfide quantum dots: growth and optical properties, p88. PhD Thesis, Università degli Studi di Roma "Tor Vergata". (2009)

[4] Walther, M. *et al.* Terahertz conductivity of thin gold films at the metal-insulator percolation transition. *Phys. Rev. B* **76**, 125408 (2007).

[5] Sihvola, A. Mixing rules with complex dielectric coefficients. *Subsurf. Sens. Technol. Appl.* **1**, 393-415 (2000).

[6] Adachi, S. *The Handbook on Optical Constants of Semiconductors: In Tables and Figures*. p17 (World Scientific, 2012).

[7] Adachi, S. *Optical constants of crystalline and amorphous semiconductors: numerical data and graphical information*. p497 (Springer Science & Business Media, 1999).

[8] Zilio, P. *et al.* Hybridization in three dimensions: a novel route toward plasmonic metamolecules. *Nano Lett.* **15**, 5200-5207 (2015).

[9] Vecchi, G., Giannini, V. & Rivas, J. G. Shaping the fluorescent emission by lattice resonances in plasmonic crystals of nanoantennas. *Phys. Rev. Lett.* **102**, 146807 (2009).

[10] Kravets, V., Schedin, F. & Grigorenko, A. Extremely narrow plasmon resonances based on diffraction coupling of localized plasmons in arrays of metallic nanoparticles. *Phys. Rev. Lett.* **101**, 087403 (2008).

[11] Zhou, W. & Odom, T. W. Tunable subradiant lattice plasmons by out-of-plane dipolar interactions. *Nat. Nanotech.* **6**, 423-427 (2011).

[12] Zou, S., Janel, N. & Schatz, G. C. Silver nanoparticle array structures that produce remarkably narrow plasmon lineshapes. *J. Chem. Phys.* **120**, 10871-10875 (2004).

[13] Koenderink, A. F. On the use of Purcell factors for plasmon antennas. *Opt. lett.* **35**, 4208-4210 (2010).

[14] Yoshie, T. *et al.* Vacuum Rabi splitting with a single quantum dot in a photonic crystal nanocavity. *Nature* **432**, 200-203 (2004).

[15] Lidzey, D. G., Bradley, D. D., Armitage, A., Walker, S. & Skolnick, M. S. Photon-mediated hybridization of Frenkel excitons in organic semiconductor microcavities. *Science* **288**, 1620-1623 (2000).

[16] Armitage, A. *et al.* Polariton-induced optical asymmetry in semiconductor microcavities. *Phys. Rev. B* **58**, 15367 (1998).

[17] Chikkaraddy, R. et al. Single-molecule strong coupling at room temperature in plasmonic nanocavities. *Nature* **535**, 127–130 (2016).

[18] Gómez, D. E., Vernon, K. C., Mulvaney, P. & Davis T. J. Surface Plasmon Mediated Strong Exciton-Photon Coupling in Semiconductor Nanocrystals. *Nano Lett.* **10**, 274-278 (2010).

[19] Trügler, A. & Hohenester, U. Strong coupling between a metallic nanoparticle and a single molecule. *Phys. Rev. B* **77**, 115403 (2008).

[20] Andreani, L. C., Panzarini, G. & Gérard, J.-M. Strong-coupling regime for quantum boxes in pillar microcavities: Theory. *Phys. Rev. B* **60**, 13276-13279 (1999).

[21] Cubukcu, E. & Capasso, F. Optical nanorod antennas as dispersive one-dimensional Fabry–Pérot resonators for surface plasmons. *Appl. Phys. Lett.* **95**, 201101 (2009).
[22] Razzari, L. *et al.* Terahertz dipole nanoantenna arrays: Resonance characteristics. *Plasmonics* **8**, 133-138 (2013).
[23] Fischer, H. & Martin, O. J. Engineering the optical response of plasmonic nanoantennas. *Opt. Express* **16**, 9144-9154 (2008).
[24] Yamamoto, Y., Tassone, F. & Cao, H. *Semiconductor cavity quantum electrodynamics*. Vol. 169 (Springer Science & Business Media, 2000).
